# 

**Published:** 1904-03-12

**Authors:** 


### CONTENTS.

**The Midwives Act** - - - - - - - - - - 417

**Medical Education and the University of London** 418

**Annotations** - - - - - - - - - - 419

**Notes and News** - - - - - - - - - - 420

**Hospital Clinics and Medical Progress—**

The Sanatorium Treatment of Consumption - - - - 421

An Unusual Case of Hernia - - - - - - - - 423

The Hypodermic Use of Cardiac Stimulants - - - - 423

PROGRESS IN DERMATOLOGY - - - - - - - 424

RHEUMATISM AND GOUT - - - - - - - - - 425

PROGRESS IN NEUROLOGY - - - - - - - - 426

**New Appliances and Things Medical** - - - 427

**Hospital Administration—**

The King's Hospital Fund - - - - - - - - 428

The League of Mercy - - - - - - - - - 430

Guy's Hospital and the Southwark Guardians - - - - 431

Hospital Meetings - - - - - - - - - 431

The "Red Cross" Light Cure and Electrical Institute - - 436

**Editor's Letter-Box** - - - - - - - 437

**The Student of Medicine** - - - - - - - 437

*NURSING SECTION.*

**Notes on News from the Nursing World—**
Queen Alexandra's Military Nursing Service—The Princess of Wales at the Evelina Hospital—Princess Christian and the Nurses of the Royal Free Hospital—Lady Dudley's Nurses' Fund—Private Nursing in Russia—A Handbook for Midwives—The Superintendent Nurse of Ladywell Workhouse—Blankets and Pillows at Netley—A Reproach to the Southampton Churches—Free or Assisted Midwifery Training—Death of a well-known Irish Nurse—Trouble at an Isolation Hospital District Nurses and Paying Patients—A New Training School—The Midwife for Midwifery Cases—District Nursing in Devonshire—Village Nursing—A Serious Deficit at Walsall—Stoke-upon-Trent Union Hospital—Short Items - - - - - - - - - - - - 317-320
**The Nursing Outlook** - - - - - - - - 321
**Lectures upon the Nursing of Mental Diseases** 322
**Private Nursing in Russia** - - - - - - 323
**Ambidexterity in the Infants' School** - - - 325
**Everybody's Opinion** - - - - - - - 325
**Appointments** - - - - - - - - - 326
**Travel Notes and Queries** - - - - - - - 326
**Echoes from the Outside World** - - - - - 327
**Notes and Queries** - - - - - - - - 328
**For Reading to the Sick** - - - - - - - 328

